# Osteoarticular Complications of Sickle Cell Disease in Bukavu: A Retrospective Multicenter Cross‐Sectional Study

**DOI:** 10.1002/hsr2.70861

**Published:** 2025-05-26

**Authors:** Rodrigue Mwenibamba Mupenda, Alexandre Nechi Nakashenyi, Daniel Safari Nteranya, Viviane Feza Bianga Viviane, Fernand Manga Opondjo, Sarah Mbula Mulasi, Didier Mbilizi Kasilembo, Christian Bisimwa Wabene, Eugene Munyantwari Akomu, Uwonda Akinja, Jean Marrie Vianney Tshimbila Kabangu

**Affiliations:** ^1^ Cliniques Universitaires de Bukavu, Faculty of Medicine Université Officielle de Bukavu Bukavu Democratic Republic of Congo; ^2^ Faculty of Medicine University of Goma Goma Democratic Republic of Congo; ^3^ Faculty of Medicine University of Mbuji Mayi Mbuji Mayi Democratic Republic of Congo; ^4^ Faculty of Medicine University of Kisangani Kisangani Democratic Republic of Congo

**Keywords:** femoral head necrosis, osteomyelitis, septic arthritis, sickle cell disease

## Abstract

**Background and Aim:**

This study aimed to investigate the epidemiological, clinical, and therapeutic aspects of osteoarticular complications in patients with sickle cell disease in Bukavu.

**Methods:**

We conducted a multicenter cross‐sectional study over 5 years, focusing on 31 patients with sickle cell disease who were admitted for osteoarticular complications in Bukavu. Data were collected from medical records and analyzed with the Statistical Package for Social Science 26 (IBM, SPSS 26).

**Results:**

We recorded 31 (6.07%) sickle cell patients with osteoarticular complications, with a mean age of 10.5 ± 5.6 years (extremes: 2–30 years). Pain, fever, and functional impotence were the most frequent reasons for consultation with 9 (29.03%) patients. Septic arthritis was the most common complication in 10 (32.26%), followed by osteonecrosis of the femoral head in 8 (25.81%), chronic osteomyelitis in 7 (22.58%) cases, and acute osteomyelitis in 4 (12.70%) patients. The femur was the most affected bone. Articular complications predominantly impacted larger joints, with the knee joint experiencing the highest rate of damage, occurring in 38.71% of cases. Treatment, including medical and surgical methods, was provided in 16 cases (51.6%). Complete healing without residual effects was achieved in 22 patients (70.96%).

**Conclusion:**

The osteoarticular complications of sickle cell disease are frequent in the city of Bukavu, and regular monitoring and early consultation would enable early diagnosis and management.

## 1 Introduction

Sickle cell disease is an autosomal recessive condition. It is caused by a mutation where a valine replaces a glutamic acid in the sixth amino acid of the hemoglobin β‐chain. This replacement leads to the production of a defective hemoglobin called hemoglobin S [1, 2].

The condition is mostly prevalent in regions with high rates of *Plasmodium falciparum* malaria cases, with high incidence of cases being found among African and Afro‐Caribbean populations [2, 3]. There is variability in the distribution of the disease from one region to another. Due to subsequent migration, there is an upsurge in the rates of sickle cell trait in northern Europe [3, 4]. It is particularly prevalent in sub‐Saharan Africa, where the prevalence sometimes exceeds 30% of the population (300,000 annual births of major sickle cell syndromes) [5]. The Democratic Republic of the Congo has one of the highest rates of sickle cell disease in sub‐Saharan Africa [6]. The evolution of the disease is marked by multiple complications, often classified as acute or chronic.

Acute complications primarily include vaso‐occlusive crises, chronic hemolytic anemia with acute exacerbations, and increased susceptibility to encapsulated bacterial infections due to functional asplenia. Chronic complications mainly affect vital organs (bones, heart, brain, liver, kidneys, lungs) [7, 8, 9, 10, 11, 12].

Bone is one of the major targets of sickle cell disease in children, mainly in the form of acute manifestations, such as vaso‐occlusive crises and infections. Chronic symptoms include osteopenia, osteoporosis, and aseptic osteonecrosis, which are more common in adults but less commonly found in children [2, 10, 13]. These complications are age‐related and mostly target bones and joints. For example, bony manifestations leading to “hand‐foot” or dactylitis syndrome are common in infants between 6 and 18 months of age. However, joint complications are most common in adults. These complications are highly invalidating, the most threatening being the femoral head necrosis [7, 13, 14].

There is seldom literature on the bone and joint complications of sickle cell disease in the Democratic Republic of the Congo despite the high prevalence of the disease. This study aimed to study the epidemiological, clinical, and therapeutic osteoarticular complications of sickle cell disease in the city of Bukavu.

## 2 Patients and Methods

We conducted a multicenter retrospective cross‐sectional study of osteoarticular complications of sickle cell disease in the city of Bukavu from January 2015 to December 2020. Data regarding patient demographics and clinical profiles were collected and analyzed. We included all the Sickle cell disease patients with a complete medical record admitted for osteoarticular complications at the University Clinics of Bukavu, Panzi General Referral Hospital, Medicure Hospital, Skyborne Hospital, and Clinique Ami Des Enfants. The data regarding our patients' epidemiological, clinical, paraclinical, therapeutic, and evolutionary information was recorded in a pre‐established Excel spreadsheet (Microsoft Corporation, Redmond, USA). Collected data were analyzed using Statistical Package for the Social Sciences (IBM, SPSS Inc.) version 26. Categorical variables are expressed as mean (±Standard Deviation), and categorical variables as frequencies.

### 2.1 Ethical Statement

The institutional review board of the Faculty of Medicine of the Official University of Bukavu approved this study. The participants willingly consented to answer our questions after being informed of the study's goals and methods. Written informed consent was obtained from each participant, and the ethical committee of each hospital approved the study. The confidentiality of the collected data was scrupulously respected.

## 3 Results

### 3.1 Epidemiological Data

In this series, the frequency of osteoarticular complications was 31 (6.07%) cases. The mean patient age was 10.5 ± 5.6 years, with extremes ranging from 2 to 30 years. The 6–10 age group was the most represented, with 16 cases (1.61%). Females were more affected by complications than males, with 16 (51.61%) cases, with a sex ratio of 1.06. Primary school patients were more represented, with 16 (51.61%) cases (Table 1).

**Table 1:** Sociodemographic data.

Variables	Records	Percentage
Age		
Mean (SD)	10.5 (5.6)	
Maximum	30	
Minimum	2	
Age class		
0–5	5	16.13%
10‐June	16	51.61%
15‐November	8	25.80%
16–20	1	3.22%
≥ 20	1	3.22%
Sex		
Female	16	51.61%
Male	15	48.61%
Education		
Illiterate	6	19.35%
Kindergarten	2	6.45%
Primary	16	51.61%
Secondary	7	22.58%
Total	31	100.00%

### 3.2 Clinical and Paraclinical Data

The HbSS form was the most common, observed in 12 cases (38.71%), while 19 cases (61.29%) had unspecified sickle cell forms.

The most frequent reasons for consultation were pain, fever, and functional impotence, with 9 (29.03%) patients (Table 2). Septic arthritis (septic osteoarthritis) was the most common complication in 10 (32.26%) patients, followed by osteonecrosis of the femoral head in 8 (25.81%) patients, chronic osteomyelitis in 7 (22.58%) cases and acute osteomyelitis in 4 (12.70%) patients. The femur was the most affected bone with 12 (38.71%) patients. The upper one‐third of the femur was the most affected bone segment, with 23 cases (74.19%). The articular complications mostly affected the big joints, the knee joint being the most damaged in 38.71% of cases (Table 3).

**Table 2:** Clinical data.

Variables	Records	Percentage
Genotype		
Unknown	19	61.29%
HbSS	12	38.71%
Follow up		
Irregular	9	29.03%
Regular	22	70.97%
Reason for consultation		
Lameness + pain + functional impotence	2	6.45%
Lameness	2	6.45%
Lameness + pain	3	9.68%
Pain + fever + functional impotence + swelling	7	22.58%
Pain + functional impotence + swelling	2	6.45%
Pain + fever	2	6.45%
Pain + fever + swelling	1	3.23%
Pain + fever + functional impotence	9	29.03%
Pain + functional impotence	1	3.23%
Pain	2	6.45%
Total	31	100.00%

**Table 3:** Paraclinical data.

Variables	Records	Percentage
Complementary examinations		
Ultrasound + biology	3	9.67%
Ultrasound + X‐ray	2	6.45%
Radiography	14	45.16%
X‐ray + biology + ultrasound	3	9.67%
X‐ray + biology	7	22.58%
X‐ray + ultrasound + biology	2	6.45%
Standard Rx imaging results		
Necrosis of the femoral head	8	25.81%
Necrosis of the humeral head	1	3.23%
Radiographic signs of arthritis	7	22.58
Periosteal reaction	3	9.68
Escrow	5	19.35
Not done	3	9.68
Fracture line	3	9.68%
Total	31	100.00%

Radiography alone was the complementary examination that established the diagnosis in 14 (45.16%) patients, followed by radiography combined with biology in 7 (22.58%) patients, and ultrasound combined with biology in 3 (9.67%) cases (Table 3).

Femoral head necrosis was most visualized on radiography in 8 (25.81%) patients followed by bone degenerative changes in 7 (22.58%) patients and sequestration in 5 (19.3%) patients (Table 4).

**Table 4:** Complications.

Variables	Records	Percentage
Complications		
Septic arthritis	10	32.26%
Tibial plateau bone infarction	1	3.23%
Acute osteomyelitis	4	12.90%
Chronic osteomyelitis	7	22.58%
Osteonecrosis of the femoral head	8	25.81%
Necrosis of the humeral head	1	3.23%
Bone damage		
Femur	12	38.71%
Humerus	9	29.03%
ulna + radius	2	6.45%
Tibia	8	25.81%
Affected bone segment		
Top 1/3	23	74.19%
Bottom 1/3	6	19.35%
1/3 medium	2	6.45%
Joint damage		
Elbow	4	12.90%
Shoulder	7	22.58%
Knee	12	38.71%
Hip	8	25.81%
Total	31	100.00%

### 3.3 Therapeutics and Evolution

Medical treatment, combined with surgical intervention when indicated, was the primary therapeutic approach utilized in 16 (51.6%) patients. Most patients underwent a regular follow‐up, consisting of weekly appointments with a pediatrician and orthopedic surgeon for the first month. At the 1‐month mark, each patient received an X‐ray of the affected area to confirm no recurrence. Nearly all patients achieved full recovery without sequelae. (Table 5).

**Table 5:** Treatment and progress.

Variables	Records	Percentage
Therapeutic approach		
Medical + surgical	16	51.61%
Medical + orthopedic	8	25.81%
Medical	7	22.58%
Occurrence of sequellae		
Healing without complications	22	70.96%
Recovery with complications	9	29.3%
Total	31	100.00%

## 4 Discussion

Sickle cell disease (SCD) encompasses a spectrum of inherited hemoglobinopathies, including homozygous sickle cell anemia (HbSS), sickle cell trait (HbAS), and compound heterozygous states such as HbSC disease and HbS/β‐thalassemia. HbSS, the most severe and predominant form in sub‐Saharan Africa, including the Democratic Republic of the Congo, significantly contributes to morbidity [1, 4]. Osteoarticular complications—namely osteonecrosis, osteomyelitis, and septic arthritis—arise from vaso‐occlusion, chronic hemolysis, and increased infection risk due to functional asplenia, disproportionately affecting children and posing substantial clinical challenges [6, 7, 8, 14] Understanding the prevalence and characteristics of these complications across SCD phenotypes is critical for interpreting study findings and guiding clinical management strategies.

### 4.1 Epidemiological Characteristics

This study identified osteoarticular complications in 31 (6.07%) of 510 SCD patients in Bukavu over a 5‐year period, with a mean age of 10.5 ± 5.6 years. The 6–10‐year age group was most affected (51.61%), and females slightly predominated (51.61%, sex ratio 0.94). These findings are consistent with regional trends of childhood onset [14]. Banza et al. reported a similar prevalence of 9.2% for osteoarticular infections among HbSS patients in Lubumbashi, with a mean age of 10.9 ± 9.5 years and a comparable sex ratio of 0.9, highlighting the severity of HbSS in the Congolese population [11]. In contrast, Yaokreh et al. observed a higher prevalence of 34.9% in HbAS patients in West Africa (mean age 7.2 ± 4.6 years), suggesting that milder forms of SCD may still result in frequent complications [15]. Chinawa et al. documented a 32.1% rate of musculoskeletal complications in 78 HbSS patients in Nigeria, attributing regional variability to geographic factors and sample size differences [16]. Fontalis et al. emphasized the diagnostic challenge of distinguishing vaso‐occlusive crises from osteomyelitis in pediatric SCD patients, underscoring the need for robust epidemiological profiling [17]. The lack of specified SCD genotypes in 61.29% of this study's cohort limits the ability to draw phenotype‐specific conclusions, a limitation also noted in prior regional studies [18].

The slight female predominance observed here contrasts with reports of higher male morbidity in SCD [19], though the autosomal recessive inheritance of SCD ensures equal incidence across genders [20]. The frequency of complications is likely driven by sickling‐induced hypoxia and thrombosis in low‐flow tissues such as bone [14, 21].

### 4.2 Clinical and Imaging Characteristics

Of the 31 patients, 12 (38.71%) were confirmed as HbSS, while 19 (61.29%) had unspecified SCD genotypes, restricting phenotype‐complication correlations. Pain, fever, and functional impairment were the primary reasons for consultation (29.03%). Septic arthritis was the leading complication (32.26%), followed by osteonecrosis of the femoral head (25.81%), chronic osteomyelitis (22.58%), and acute osteomyelitis (12.70%). The knee was the most frequently affected joint (38.71%), followed by the hip (25.81%), with the proximal third of the bone involved in 74.19% of cases. Adesina et al. reported a 22% incidence of osteonecrosis in 6237 SCD patients (predominantly HbSS), with a median onset at 27 years, more common in severe phenotypes, reflecting its chronic burden [22]. Milner et al.'s longitudinal study of 2590 HbSS patients identified a baseline femoral head osteonecrosis prevalence of 9.8%, with an annual incidence of 2%–4.5%, indicating a progressive risk [23]. In contrast, the predominance of septic arthritis in this study may be attributed to the younger cohort (mean age 10.5 years) and regional infection risks, as supported by Nathani and Samal, who linked septic arthritis in SCD to osteomyelitis and joint damage in children [24]. Fontalis et al. noted that septic arthritis often coexists with bone infarction in HbSS patients, complicating diagnosis [17]. The knee's prominence over the hip in this study, differing from the hip‐focused osteonecrosis in Adesina et al. and Milner et al., may result from reliance on radiography (used in 45.16% of cases) or local infection patterns [22, 23]. Recent reviews underscore the utility of advanced imaging, such as MRI, in differentiating osteoarticular pathologies in SCD, highlighting a limitation of this study's diagnostic approach [25, 26].

### 4.3 Therapeutic Approaches and Outcomes

Treatment strategies integrated medical and surgical interventions in 51.6% of cases, with medical‐only and orthopedic approaches employed in 22.58% and 25.81% of cases, respectively. Antibiotics and analgesics targeted infections and pain, while surgical interventions, such as sequestrectomy, were reserved for cases where conservative treatments failed [27]. Banza et al. reported a lower surgical rate of 17.1% in HbSS patients, potentially reflecting less severe infections in Lubumbashi [11]. Conversely, Gouveia et al. found that 53% of HbSS cases required surgery due to resistant infections, aligning with the combined approach in this study [28]. Healing without sequelae occurred in 70.96% of patients, while 29.03% experienced complications, including joint stiffness and chronic osteomyelitis. Djomo Tamchom et al. reported a similar complication rate of 23.4% in HbSS patients, with a 3.2% mortality rate not observed here, possibly due to the smaller sample size (31 vs. 124) [29]. Mohzari et al. advocate early antibiotic adjustments to improve outcomes in pediatric SCD‐related osteoarticular infections, supporting the high success rate observed in this study [30].

The elevated healing rate reflects the efficacy of the treatment approach; however, the absence of specified SCD genotypes and reliance on radiography may underestimate complication severity. Tailored therapeutic strategies, considering infection profiles and SCD phenotypes, are critical for optimizing orthopedic outcomes [30, 31].

### 4.4 Implications and Future Directions

Osteoarticular complications represent a significant burden for SCD patients in Bukavu, particularly among children. The predominance of septic arthritis over osteonecrosis, differing from the findings of Adesina et al. and Milner et al., may reflect diagnostic limitations or regional environmental factors [22, 23]. Banza et al.'s regional data highlight similar challenges in the Congo, emphasizing the need for standardized genotyping [11]. Regular monitoring and early intervention could mitigate morbidity [7]. Future studies should prioritize comprehensive genotyping of all patients and the use of advanced diagnostics, such as MRI, to better delineate SCD phenotype‐specific outcomes, thereby improving clinical relevance and comparability [17, 25].

## 5 Conclusion

The osteoarticular complications of sickle cell disease are prevalent in Bukavu, necessitating regular monitoring and early consultation to facilitate timely diagnosis and management. A large‐scale study with a well‐defined phenotype for each enrolled case is essential to elucidate the association between SCD phenotypes and osteoarticular complications.

### 5.1 Limitations of the Study

- Lack of a control group limits the ability to compare the prevalence and severity of osteoarticular complications with non‐SCD populations or different treatment protocols.
- Nearly 61% of the patients had unspecified forms of SCD, which could affect the generalizability and interpretation of the results.
- Reliance primarily on radiography for diagnosis, with limited use of other diagnostic modalities, might have affected the comprehensiveness of the clinical assessment.

## Author Contributions

**Rodrigue Mwenibamba Mupenda:** conceptualization, methodology, and validation. **Alexandre Nechi Nakashenyi:** writing – review and editing, writing – original draft, conceptualization, and methodology. **Daniel Safari Nteranya:** software, writing – original draft, writing – review and editing, visualization, validation, resources, and methodology. **Viviane Feza Bianga Viviane:** writing – original draft, writing – review and editing, validation. **Fernand Manga Opondjo:** writing – original draft, writing – review and editing, validation. **Sarah Mbula Mulasi:** conceptualization, methodology, and investigation. **Didier Mbilizi Kasilembo:** writing – original draft, writing – review and editing. **Christian Bisimwa Wabene:** writing – original draft, writing – review and editing, validation, resources, and software. **Eugene Munyantwari Akomu:** writing – review and editing, writing – original draft, and validation. **Uwonda Akinja:** writing – review and editing, writing – original draft, and validation. **Jean Marrie Vianney Tshimbila Kabangu:** writing – review and editing, writing – original draft, supervision, and project administration.

## Conflicts of Interest

The authors declare no conflicts of interest.

## Transparency Statement

The lead author Rodrigue Mupenda Mwenibamba affirms that this manuscript is an honest, accurate, and transparent account of the study being reported; that no important aspects of the study have been omitted; and that any discrepancies from the study as planned (and, if relevant, registered) have been explained.

## Data Availability

The data of this study are available upon request to the corresponding author.

## References

[1] P. L. Kavanagh , T. A. Fasipe , and T. Wun , “Sickle Cell Disease: A Review,” Journal of the American Medical Association 328, no. 1 (July 2022): 57–68. 35788790 10.1001/jama.2022.10233

[2] E. Conway O'Brien , S. Ali , and T. Chevassut , “Sickle Cell Disease: An Update,” Clinical Medicine (London, England) 22, no. 3 (May 2022): 218–220. 35584832 10.7861/clinmed.2022-0143 PMC9135093

[3] GBD 2021 Sickle Cell Disease Collaborators , Global, Regional, and National Prevalence and Mortality Burden of Sickle Cell Disease, 2000–2021: A Systematic Analysis From the Global Burden of Disease Study 2021,” Lancet Haematology 10, no. 8 (2023): e585–e599, https://pubmed.ncbi.nlm.nih.gov/37331373/. 37331373 10.1016/S2352-3026(23)00118-7 PMC10390339

[4] O. Adigwe , S. Onoja , and G. Onavbavba , “A Critical Review of Sickle Cell Disease Burden and Challenges in Sub‐Saharan Africa,” Journal of Blood Medicine 14 (2023): 367–376, https://pubmed.ncbi.nlm.nih.gov/37284610/. 37284610 10.2147/JBM.S406196 PMC10239624

[5] D. Bachir , F. Zemirline , A. Niakate , E. Cabaret , and F. Galactéros , “Drépanocytose: Le Médecin Du Travail, Relais Essentiel Dans L'Information Et La Prise En Charge,” Archives des Maladies Professionnelles et de L'Environnement 76, no. 4 (2015): 373–385, https://linkinghub.elsevier.com/retrieve/pii/S1775878515000843.

[6] B. Ranque , R. Kitenge , and D. Ndiaye , et al., “Estimating the Risk of Child Mortality Attributable to Sickle Cell Anaemia in Sub‐Saharan Africa: A Retrospective, Multicentre, Case‐Control Study,” Lancet Haematology 9, no. 3 (2022): e208–e216, https://pubmed.ncbi.nlm.nih.gov/35240076/. 35240076 10.1016/S2352-3026(22)00004-7

[7] A. Diakité , A. Dembélé , M. Cissé , et al., “Complications Ostéoarticulaires De La Drépanocytose Au Département De Pédiatrie Du CHU Gabriel Touré,” Health Sciences and Disease 20, no. 4 (July 2019), https://www.hsd-fmsb.org/index.php/hsd/article/view/1474.

[8] M. R. DeBaun , L. C. Jordan , A. A. King , et al., “American Society of Hematology 2020 Guidelines for Sickle Cell Disease: Prevention, Diagnosis, and Treatment of Cerebrovascular Disease in Children and Adults,” Blood Advances 4, no. 8 (April 2020): 1554–1588. 32298430 10.1182/bloodadvances.2019001142 PMC7189278

[9] “Acute and Chronic Bone Complications of Sickle Cell Disease,” UpToDate, accessed February 13, 2024, https://www.uptodate.com/contents/acute-and-chronic-bone-complications-of-sickle-cell-disease.

[10] E. Hegeman , T. Bates , T. Lynch , and M. Schmitz , “Osteomyelitis in Sickle Cell Anemia: Does Age Predict Risk of Salmonella Infection?,” Pediatric Infectious Disease Journal 42, no. 8 (2023): e262–e267, https://pubmed.ncbi.nlm.nih.gov/37079601/. 37079601 10.1097/INF.0000000000003937

[11] M. Banza , N. Kapessa , A. Mukakala , et al., “Osteoarticular Infections in Patients With Sickle Cell Disease in Lubumbashi: Epidemiological Study Focusing on Etiology and Management,” Pan African Medical Journal 38 (January 2021): 77, https://pubmed.ncbi.nlm.nih.gov/33889243/. 33889243 10.11604/pamj.2021.38.77.21484 PMC8033183

[12] S. Diop and F. Pirenne , “Transfusion and Sickle Cell Anemia in Africa,” Transfusion Clinique Et Biologique: Journal De La Societe Francaise De Transfusion Sanguine 28, no. 2 (May 2021): 143–145. 33515732 10.1016/j.tracli.2021.01.013

[13] I. Benenson and S. Porter , “Sickle Cell Disease: Bone, Joint, Muscle, and Motor Complications,” Orthopedic Nursing 37, no. 4 (2018): 221–227, https://pubmed.ncbi.nlm.nih.gov/30028422/. 30028422 10.1097/NOR.0000000000000464

[14] M. Brown , D. Anheyer , and C. R. Morris , “Special Issue: Pediatric Pain and Sickle Cell Disease,” Complementary Therapies in Medicine 71 (December 2022): 102880. 36031024 10.1016/j.ctim.2022.102880

[15] J. Yaokreh , H. Thomas , P. Ekobo , G. Kouamé , B. Kouamé , and O. Ouattara , “Haematogenous Osteoarticular Infections in Paediatric Sickle Cell Trait Patients: A Reality in a Tertiary Centre in West Africa,” African Journal of Paediatric Surgery 18, no. 1 (March 2021): 62–66, https://pubmed.ncbi.nlm.nih.gov/33595545/. 33595545 10.4103/ajps.AJPS_114_20 PMC8109757

[16] J. Chinawa , B. Chukwu , A. Ikefuna , and I. Emodi , “Musculoskeletal Complications Among Children With Sickle Cell Admitted in University of Nigeria Teaching Hospital Ituku ‐ Ozalla Enugu: A 58 Month Review,” Annals of Medical and Health Sciences Research 3, no. 4 (2013): 564–567, https://pubmed.ncbi.nlm.nih.gov/24380009/. 24380009 10.4103/2141-9248.122110 PMC3868124

[17] A. Fontalis , K. Hughes , M. P. Nguyen , et al., “The Challenge of Differentiating Vaso‐Occlusive Crises From Osteomyelitis in Children With Sickle Cell Disease and Bone Pain: A 15‐year Retrospective Review,” Journal of Children's Orthopaedics 13, no. 1 (February 2019): 33–39, https://www.ncbi.nlm.nih.gov/pmc/articles/PMC6376437/. 10.1302/1863-2548.12.180094 PMC6376437 30838073

[18] A. M. Thomson , T. A. McHugh , A. P. Oron , et al., “Global, Regional, and National Prevalence and Mortality Burden of Sickle Cell Disease, 2000–2021: A Systematic Analysis From the Global Burden of Disease Study 2021,” Lancet Haematology 10, no. 8 (August 2023): e585–e599, https://www.thelancet.com/journals/lanhae/article/PIIS2352-3026(23)00118-7/abstract. 37331373 10.1016/S2352-3026(23)00118-7 PMC10390339

[19] G. Ceglie , M. Di Mauro , and I. Tarissi De Jacobis , et al., “Gender‐Related Differences in Sickle Cell Disease in a Pediatric Cohort: A Single‐Center Retrospective Study,” Frontiers in Molecular Biosciences 6 (2019): 140, https://pubmed.ncbi.nlm.nih.gov/31867340/. 31867340 10.3389/fmolb.2019.00140 PMC6906547

[20] M. Kwarteng‐Siaw , V. Paintsil , C. Korankye Toboh , A. Owusu‐Ansah , and N. S. Green , “Assessment of Transition Readiness in Adolescents With Sickle Cell Disease and Their Caretakers, A Single Institution Experience,” International Journal of Hematology Research 3, no. 1 (2017): 171–179. 30035240 10.17554/j.issn.2409-3548.2017.03.47 PMC6054488

[21] V. Paintsil , E. Amuzu , I. Nyanor , et al., “Establishing a Sickle Cell Disease Registry in Africa: Experience From the Sickle Pan‐African Research Consortium, Kumasi‐Ghana,” Frontiers in Genetics 13 (February 2022): 802355, https://pubmed.ncbi.nlm.nih.gov/35281803/. 35281803 10.3389/fgene.2022.802355 PMC8908904

[22] O. Adesina , A. Brunson , T. H. M. Keegan , and T. Wun , “Osteonecrosis of the Femoral Head in Sickle Cell Disease: Prevalence, Comorbidities, and Surgical Outcomes in California,” Blood Advances 1, no. 16 (July 2017): 1287–1295. 29296770 10.1182/bloodadvances.2017005256 PMC5728545

[23] P. F. Milner , A. P. Kraus , J. I. Sebes , et al., “Sickle Cell Disease as a Cause of Osteonecrosis of the Femoral Head,” New England Journal of Medicine 325, no. 21 (November 1991): 1476–1481. 1944426 10.1056/NEJM199111213252104

[24] H. R. Nathani and S. Samal , “Septic Arthritis Associated With Hip Joint Subluxation and Epiphyseal Plate Deformation as a Sequala of Sickle Cell Anemia,” Cureus 15, no. 11: e48103, https://www.ncbi.nlm.nih.gov/pmc/articles/PMC10691398/. 10.7759/cureus.48103 PMC10691398 38046768

[25] V. Kosaraju , A. Harwani , S. Partovi , et al., “Imaging of Musculoskeletal Manifestations in Sickle Cell Disease Patients,” British Journal of Radiology 90, no. 1073 (May 2017): 20160130, https://academic.oup.com/bjr/article/7476115. 28281830 10.1259/bjr.20160130 PMC5605094

[26] R. Vaishya , A. K. Agarwal , E. O. Edomwonyi , and V. Vijay , “Musculoskeletal Manifestations of Sickle Cell Disease: A Review,” Cureus 7 (October 2015): e358, http://www.cureus.com/articles/3152-musculoskeletal-manifestations-of-sickle-cell-disease-a-review. 26623213 10.7759/cureus.358 PMC4659689

[27] D. Petek , D. Hannouche , and D. Suva , “Osteonecrosis of the Femoral Head: Pathophysiology and Current Concepts of Treatment,” EFORT Open Reviews 4, no. 3 (March 2019): 85–97, https://eor.bioscientifica.com/view/journals/eor/4/3/2058-5241.4.180036.xml. 30993010 10.1302/2058-5241.4.180036 PMC6440301

[28] C. Gouveia , M. Duarte , S. Norte , et al., “Osteoarticular Infections in Paediatric Sickle Cell Disease: In the era of Multidrugresistant Bacteria,” British Journal of Haematology 189, no. 4 (2020), 10.1111/bjh.16568. 32150291

[29] D. Djomo Tamchom , C. Eposse Ekoube , B. Essola , S. Nga Nomo , F. S. Benghiat , and L. Van Obbergh , “Incidence of Post‐Operative Complications and Factors Influencing Their Occurrence in Patients With Sickle Cell Disease in a Low‐Income Country: A Case Study of Cameroon,” Journal of Clinical Medicine 11, no. 3 (January 2022): 780. 35160234 10.3390/jcm11030780 PMC8836843

[30] Y. A. Mohzari , R. Alshuraim , S. M. B. Asdaq , et al., “Early Oral Switch to Combined Cefixime Therapy for Management of Osteoarticular Infections in Pediatric Sickle Cell Disease Patients: A Descriptive Analysis,” Journal of Infection and Public Health 15, no. 1 (January 2022): 1–6. 34852307 10.1016/j.jiph.2021.11.006

[31] A. Almeida and I. Roberts , “Bone Involvement in Sickle Cell Disease,” British Journal of Haematology 129, no. 4 (May 2005): 482–490. 15877730 10.1111/j.1365-2141.2005.05476.x

